# Age‐related remodeling of small arteries is accompanied by increased sphingomyelinase activity and accumulation of long‐chain ceramides

**DOI:** 10.14814/phy2.12015

**Published:** 2014-05-28

**Authors:** Jacqueline Ohanian, Aiyin Liao, Simon P. Forman, Vasken Ohanian

**Affiliations:** 1 Institute of Cardiovascular Sciences, Manchester Academic Health Sciences Centre, University of Manchester, Manchester, UK

**Keywords:** Aging, sphingolipids, vascular remodeling

## Abstract

The structure and function of large arteries alters with age leading to increased risk of cardiovascular disease. Age‐related large artery remodeling and arteriosclerosis is associated with increased collagen deposition, inflammation, and endothelial dysfunction. Bioactive sphingolipids are known to regulate these processes, and are also involved in aging and cellular senescence. However, less is known about age‐associated alterations in small artery morphology and function or whether changes in arterial sphingolipids occur in aging. We show that mesenteric small arteries from old sheep have increased lumen diameter and media thickness without a change in media to lumen ratio, indicative of outward hypertrophic remodeling. This remodeling occurred without overt changes in blood pressure or pulse pressure indicating it was a consequence of aging per se. There was no age‐associated change in mechanical properties of the arteries despite an increase in total collagen content and deposition of collagen in a thickened intima layer in arteries from old animals. Analysis of the sphingolipid profile showed an increase in long‐chain ceramide (C14–C20), but no change in the levels of sphingosine or sphingosine‐1‐phosphate in arteries from old compared to young animals. This was accompanied by a parallel increase in acid and neutral sphingomyelinase activity in old arteries compared to young. This study demonstrates remodeling of small arteries during aging that is accompanied by accumulation of long‐chain ceramides. This suggests that sphingolipids may be important mediators of vascular aging.

## Abstract (precis)

In this study, we have investigated remodeling of small arteries in a large animal model of aging, the sheep. We show that there is age‐related formation of neointima and increased collagen deposition that is accompanied by changes in sphingolipid metabolism resulting in ceramide accumulation in the tissues. These are the first data implicating sphingolipids as important mediators of vascular aging in small arteries. Given that aging is a major risk factor for cardiovascular disease, our study opens a new area for further research into the mechanisms that underlie vascular remodeling in aging.

## Introduction

Cardiovascular disease increases exponentially with age and is the major cause of morbidity and mortality in the elderly. During aging, changes in the structure and function of large and small arteries results in a less compliant vasculature that is less able to respond to vasodilator signals. These alterations lead to increased systolic pressure and pulse width, changes in peripheral vascular resistance, development of hypertension, greater susceptibility to atherosclerotic plaque formation, and poor autoregulation of blood flow to end organs (Lakatta and Levy 2003; McEniery et al. 2007, 2010; Wang et al. 2010). The age‐related changes that occur in the wall of large conduit arteries and the mechanisms underlying such changes are well documented (Lakatta and Levy 2003; Ungvari et al. 2010; Graham et al. 2011). However, our understanding of the changes that occur in small arteries is less clear.

Studies in aged rats have shown stiffening and hypertrophic remodeling of small mesenteric resistance arteries (Moreau et al. 1998; Adrian et al. 2004; Laurant et al. 2004; Briones et al. 2007) that correlates with increased pulse width (Moreau et al. 1998), an important predictor of adverse cardiovascular events in the elderly (McEniery et al. 2010). In addition to smooth muscle cell hypertrophy, there is increased collagen content and elastin structure changes in the artery wall with age (Briones et al. 2007) and these extracellular matrix changes are thought to contribute to the increased stiffness (Briones et al. 2007). Although whether such changes lead to altered distensibility is uncertain with one study reporting an age‐related decrease (Briones et al. 2007) and others no difference (Adrian et al. 2004; Laurant et al. 2004; Mandalà et al. 2012) in this parameter in small resistance arteries from rodent models of aging.

The molecular mechanisms that contribute to the age‐associated remodeling in resistance arteries are still unclear. Studies in endothelial cells isolated from rat aorta have shown increases in ceramide levels in cells from old animals compared to young (Smith et al. 2006). Ceramide is a sphingolipid implicated in cell senescence, apoptosis, and fibrosis (Hannun and Obeid 2008) and alterations in sphingolipid levels and metabolism have been described in aging and cardiovascular disease (Alewijnse and Peters 2008; Nikolova‐Karakashian et al. 2008). Additionally, in model organisms such as yeast and Drosophila, a direct link between sphingolipid genes and longevity has been described (D'Mello et al. 1994; Guillas et al. 2001; Schorling et al. 2001; Rao et al. 2007). In mammals, including humans, increased sphingomyelinase activity and the subsequent generation of ceramide is associated with aging (Lecka‐Czernik et al. 1996; Lightle et al. 2000; Smith et al. 2006; Nikolova‐Karakashian et al. 2008; Patschan et al. 2008; Venable and Yin 2009). The major classes of sphingomyelinase are acid and neutral, and although both are activated by inflammatory cytokines and oxidative stress (Hannun and Obeid 2008) resulting in increased ceramide production; some differences in response to their activation are observed. For instance, increased acid sphingomyelinase (A‐SMase) activity appears to be associated with cell senescence and premature aging (Lecka‐Czernik et al. 1996; Patschan et al. 2008; Venable and Yin 2009). While increased neutral sphingomyelinase (N‐SMase) activity is the major source of ceramide in aged vascular and nonvascular cells and tissues (Lightle et al. 2000; Smith et al. 2006; Nikolova‐Karakashian et al. 2008). Chronic low‐grade inflammation, driven in part by increased levels of inflammatory cytokines (Ungvari et al. 2010), and oxidative stress (Franceschi et al. 2000) are associated with vascular aging suggesting that sphingolipids may regulate the age‐related changes that occur in the artery wall. Certainly, ceramide regulates cellular aging processes such as apoptosis, autophagy, and senescence (Obeid and Hannun 2003) and the levels of this lipid are raised in tissues from aged rats (Lightle et al. 2000) and mice (Hernández‐Corbacho et al. 2011). In addition to their role in aging, sphingolipids are also profibrotic. For instance, ceramide promotes collagen production in dermal fibroblasts (Sato et al. 2003) and lung tissue (Dhami et al. 2010) and its metabolite sphingosine‐1‐phosphate (S1P) stimulates tissue inhibitor of metalloprotease (TIMP) expression leading to inhibition of matrix metalloproteases (MMP) and reduced collagen degradation (Yamanaka et al. 2004). Although whether sphingolipids are involved in collagen production in the vessel wall is not known. Collectively these studies suggest that sphingomyelinases by increasing production of ceramide may be involved in the signaling pathways leading to artery remodeling in aging.

To date evidence for age‐related changes in small arteries has been reported mainly from rodent models of aging. But there have been few studies in large animal models that have greater longevity and where heart rate and blood pressure more closely reflect human hemodynamics. Sheep have been used extensively as a large animal model to study remodeling and changes in arterial function in hypoxia, pulmonary hypertension, pregnancy, and during maturation (Akopov et al. 1998; Williams and Pearce 2006; Herrera et al. 2010; Xiao et al. 2010). They are also an established model of aging (Dibb et al. 2004) and a recent report showed an age‐related increase in aortic stiffness accompanied by increased deposition of collagen and elastic fibers (Graham et al. 2011) changes similar to those seen in humans (Lakatta and Levy 2003; Greenwald 2007). However, whether remodeling of small arteries also occurs is not known. Approximately a quarter of cardiac output is directed to the intestine, accordingly mobilization of splanchnic blood to other areas of the body is integral to maintaining exercise capacity (Rowell 1974; Flamm et al. 1990). Accordingly, we investigated age‐associated changes in the structure and function of mesenteric arteries in this large animal model of aging. We show that there is outward hypertrophic remodeling (increased lumen diameter and wall cross‐sectional area), neointima formation, and increased collagen content, but no increase in stiffness or change in distensibility in sheep mesenteric small arteries with age. In addition, ceramide levels were increased in old arteries compared to young and this was paralleled by increased sphingomyelinase activity suggesting a role for altered sphingolipid metabolism in mesenteric small artery remodeling in aging.

## Methods

### Animals and tissues

The investigation was carried out in accordance with *Guide for the Care and Use of Laboratory Animals* published by the U.S. National Institutes of Health (NIH Publication No. 85‐23, revised 1996), The University of Manchester Animal Experimentation Guidelines, and the U.K. Animals (Scientific Procedures) Act 1986. Experiments were performed with the approval of the Review Board of the University of Manchester and the Home Office.

Young (18–24 months) and old (>8 years; representing sexual maturity and the last quintile of life respectively) female sheep (*Ovis aries*) were used in the study. Heart rate was determined from surface electrocardiograms and indirect blood pressure measurements were made from the coccygeal artery using tail cuff plethysmography in conscious standing animals as described previously (Horn et al. 2012).

Following in vivo measurements animals were killed by intravenous injection of pentobarbitone (200 mg/kg; Dibb et al. 2004). A portion of the small intestine was removed and mesenteric small arteries (<400 *μ*m internal diameter) were immediately dissected and placed into ice‐cold physiological salt solution (PSS contained [in mmol/L] 119 NaCl, 4.7 KCl, 25 NaHCO_3_, 1.17 MgSO_4_.7H_2_O, 1.18 KH_2_PO_4_, 0.026 K_2_EDTA, 5.5 glucose, and 1.6 CaCl_2_.2H_2_O). Arteries were then prepared for pressure myography, histology, or sphingomyelinase activity assay as detailed below.

#### Pressure myography

Vascular function was determined by pressure myography (Living Systems, Burlington, VT) as described previously (Clarke et al. 2008). Segments of small artery were dissected immediately proximal to the intestinal wall in order to ensure samples from the same branch order and anatomical area were taken from both young and aged animals. Arteries were tied onto glass cannulae in the arteriograph chamber and after equilibration at 20 mmHg and 37°C in PSS pH 7.4, gassed with 5% CO_2_ in air), intraluminal pressure was raised to 70 mmHg and the vessel left to stabilize for 15 min before addition of 50 mmol/L KPSS (high potassium physiological salt solution, molar substitution with NaCl) to test viability. Only artery segments that showed a greater than 50% constriction to KPSS were included in the study of reactivity. Cumulative concentration response curves were constructed to noradrenaline (NA; 0.1–100 *μ*mol/L). Following washout and after 30‐min equilibration, vessels were constricted with NA 10 *μ*mol/L and cumulative concentration response curves to acetylcholine (ACh; 0.01–100 *μ*mol/L) were constructed. Lumen diameter was measured 2 min following the addition of agonist immediately before addition of the next concentration. Following measurement of responses to NA and ACh, artery segments were incubated for 30 min in calcium‐free PSS containing 2 mmol/L EGTA to determine maximal passive diameter at 70 mmHg (dia_passive_). Myogenic tone was calculated as: (dia_passive_ − dia_active_)/dia_passive_, where dia_active_ is the diameter at 70 mmHg in PSS. The vasoconstriction response to NA was calculated as: (dia_passive_ − dia_response_)/dia_passive_, where dia_response_ is the diameter at any given drug concentration. The vasodilation response to ACh was calculated as: (dia_agonist_ − dia_base_)/(dia_passive_ − dia_base_), where dia_agonist_ is the diameter at any given ACh concentration and dia_base_ is the NA preconstricted diameter. Any vessels unable to maintain pressure were excluded from the study.

Structural and mechanical properties of mesenteric small artery (MSA) segments were determined as previously described (Izzard et al. 2006). Briefly, following incubation for 30 min in calcium‐free PSS containing 2 mmol/L EGTA to ensure maximal relaxation intraluminal pressure was decreased to 5 mmHg (unstressed diameter) then increased in steps to 10, 20, 40, 60, 100, 140, and 180 mmHg. Lumen diameter and wall thickness were measured at each pressure step and the following structural parameters were measured:

The wall/lumen ratio was calculated as WT/*D* × 100, where WT is the wall thickness and *D* is lumen diameter.
Wall cross‐sectional area (CSA) was calculated as: CSA = *p* [*D* + 2WT/2]^2^ − *p* (*D*/2)^2^.
Wall stress (s) = *P* × *D*/2WT, where *P* is pressure and 1 mmHg = 1334 dyn/cm^2^.
Wall strain (e) = (*D* − *D*_0_)/*D*_0_, where *D*_0_ is the lumen diameter at 5 mmHg.

The stress–strain data for each artery was fitted to the curve *σ *= *σ*_0_.e^*β*.*ε*^, where *σ*_0_ is the stress at 5 mmHg and *β* is the slope of the tangential elastic modulus versus stress relation and is an index of distensibility; the higher the value of *β*, the lower the arterial distensibility (Izzard et al. 2006; Briones et al. 2007).

### Histological analysis of collagen and elastin

Segments of MSA were dissected, fixed under passive conditions in 4% paraformaldehyde, and placed into 75% ethanol before embedding in paraffin blocks. Histology was performed on 10‐*μ*m sections of MSA. Tissue was stained with hematoxylin and eosin. Collagen was stained with picrosirius red (PSR) and visualized using polarized light microscopy, elastin was stained on a consecutive section with Miller's reagent (Graham and Trafford 2007). Total collagen or elastin content is expressed as percentage collagen or elastin containing pixels per tissue section (Briones et al. 2007; Graham and Trafford 2007; Horn et al. 2012).

#### Immunofluorescence

Immunofluorescence was performed on 5‐*μ*m sections of MSA. Sections were coincubated with *α*‐smooth muscle actin (*α*‐sma, Clone 1A4) and von Willebrand Factor (vWF) antibodies overnight at 4°C. Negative controls were coincubated with rabbit and mouse IgG at the same concentrations as the corresponding primary antibody. Sections were then incubated with the appropriate Alexa Fluor‐488 or Rhodamine Red X conjugated secondary antibodies and Hoechst nuclear stain. The sections were observed and photographed using a Leica CTR5000 microscope.

#### Tissue homogenate preparation

Arteries were homogenized in Tris‐TX‐100 buffer (25 mmol/L Tris pH 7.4, 5 mmol/L EDTA, 0.2% Triton X‐100, protease inhibitors [Complete mini‐tab; Roche, Welwyn Garden City, UK], 1 mmol/L sodium orthovanadate, 200 *μ*mol/L sodium pyrophosphate) on ice. The homogenate was incubated at 4°C for 30 min, centrifuged at 800*g* for 10 min at 4°C to pellet nuclei and cell debris and the protein concentration of the supernatant determined by Bradford assay. The sample volume was adjusted with Tris‐TX‐100 buffer to a final concentration of 1 mg/mL. An aliquot was removed for sphingomyelinase assay and to the remainder Laemmli sample buffer was added and the sample stored at −20°C.

#### In vitro sphingomyelinase assays and lipid measurements

Sphingomyelinase activity was measured in freshly prepared homogenates using NBD‐C6‐Sphingomyelin (SM) as described previously (Loidl et al. 2002; Ohanian et al. 2012). For N‐SMase activity, tissue homogenate (25‐*μ*g protein) was added to 100 *μ*L of reaction mixture containing; 100 mmol/L Tris pH 7.4, 10 mmol/L MgCl_2_, 0.2% Triton X‐100, 10 mmol/L dithiothreitol, 100 *μ*mol/L NBD‐C6‐SM, and 100 *μ*mol/L phosphatidylserine. For A‐SMase activity, tissue homogenate (25‐*μ*g protein) was added to 100 *μ*L of reaction mix containing; 0.25 mol/L sodium‐acetate pH 5.0, 1 mmol/L EDTA, 0.1% Triton X‐100, and 100 *μ*mol/L NBD‐C6‐SM. Following 30‐min incubation at 37°C, reactions were terminated by the addition of 1 mL chloroform:methanol (2:1 v:v) and 200 *μ*L dH_2_O for phase separation. The lower organic phase was dried under O_2_‐free N_2_ gas and resuspended in 15 *μ*L chloroform:methanol (2:1 v:v). Samples and NBD‐C6‐ceramide standard were resolved by thin layer chromatography and the fluorescent lipid detected using an Alpha‐Innotech Imager (ProteinSimple, Santa Clara, CA). NBD‐C6‐ceramide was identified by the cochromatographed standard and quantified by densitometry using AlphaEaseFC software (ProteinSimple). For lipid analysis by mass spectrometry, MSA were homogenized in RIPA buffer and homogenate containing 1‐mg protein was analyzed for ceramide, dihydroceramide, and sphingoid bases by tandem liquid chromatography/mass spectrometry (Bielawski et al. 2010). Lipid levels were normalized to cellular protein. Lipid analysis was carried out by the Lipidomics Core Facility at the Medical University of South Carolina.

#### Data analysis

Data are expressed as means ± SEM and the number of individual animals used for each experiment is given in the figure legends. Statistical analysis was performed by Student's *t*‐test between individual groups, or one‐way analysis of variance (ANOVA) followed by Bonferroni posttest for multiple comparisons using GraphPad Prism software, (GraphPad Software Inc., La Jolla, CA) *P *<**0.05 was considered statistically significant.

#### Materials

Noradrenaline and ACh were purchased from Sigma (Poole, Dorset, UK). NBD‐C6‐SM and NBD‐C6‐ceramide were from Molecular Probes (Invitrogen, Life Technologies Ltd, Paisley, UK). Antibodies used were monoclonal *α*‐smooth muscle actin (Sigma, UK) and polyclonal von Willebrand Factor (Dako, Cambridge, UK) and negative controls; rabbit and mouse IgG (Santa Cruz Biotechnology, Insight Biotechnology Ltd, Wembley, UK). Alexa Fluor‐488 or Rhodamine Red X conjugated secondary antibodies were from Jackson Laboratories (Scientific Ltd, Newmarket, UK). Salts and chemicals were from Sigma UK.

## Results

There were no differences in body weight, heart rate, mean arterial pressure, or pulse pressure between young and old animals (Table 1).

**Table 1.** Characteristics of young and old sheep

	Young (<18 months)	Old (>8 years)
Number of sheep	9	9
Body weight (kg)	32 ± 2	35 ± 3
Mean arterial pressure (mmHg)	98 ± 3	100 ± 3
Pulse pressure (mmHg)	49 ± 2	52 ± 4
Heart rate (bpm)	111 ± 8	125 ± 8

Data are mean ± SEM.

### Small artery structure

**Figure 1.**
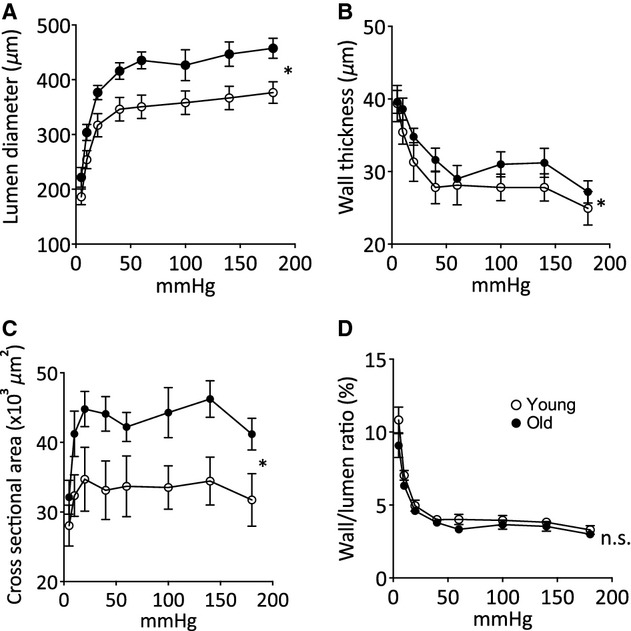
Mesenteric small artery lumen diameter and wall characteristics in response to in vitro pressure changes. (A) Internal diameter–intraluminal pressure relationship, (B) wall thickness–intraluminal pressure relationship, (C) wall cross‐sectional area–intraluminal pressure relationship, (D) wall/lumen ratio–intraluminal pressure relationship in mesenteric small arteries from young (open circles) and old (closed circles) sheep in calcium‐free physiological salt solution. Data are expressed as mean ± SEM from individual arteries from eight young and five old animals, *P <**0.05 by ANOVA.

The lumen diameter of MSA from old animals was increased across the entire pressure range, compared with young animals (Fig. 1A). The MSA from old animals also showed increased pressure–wall thickness and pressure–cross‐sectional area relationships, compared with young animals (Fig. 1B and C). However, there was no difference in the pressure–wall/lumen ratio relationship between MSA from old and young animals (Fig. 1D), demonstrating outward hypertrophic remodeling of the arteries with age (Mulvany 1999).

### Collagen and elastin content of mesenteric small arteries

**Figure 2.**
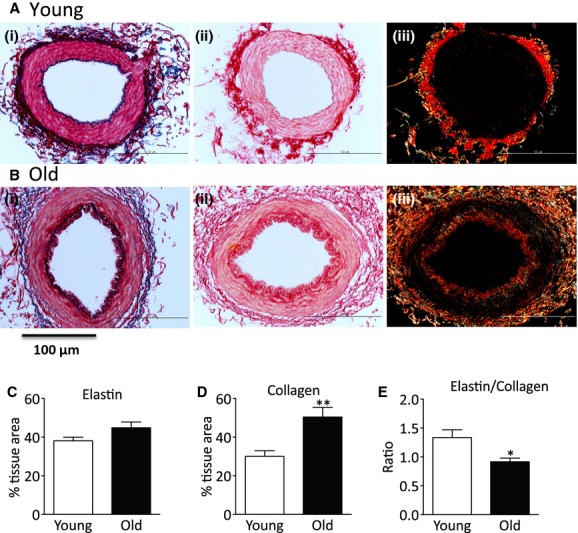
Collagen and elastin content of mesenteric small arteries. (Ai and Bi) Representative sections of mesenteric small arteries (MSA) stained with Miller reagent for elastin, (Aii, Aiii and Bii, Biii) representative sections of MSA stained with picrosirius red for collagen illuminated with (ii) normal and (iii) polarized light. Upper panels MSA from young and lower panels MSA from old animals. Quantitation of (C) elastin, (D) collagen, and (E) elastin/collagen ratio. Data are expressed as mean ± SEM from individual arteries from seven young and seven old animals, *P <**0.05 by Student's *t*‐test.

**Figure 3.**
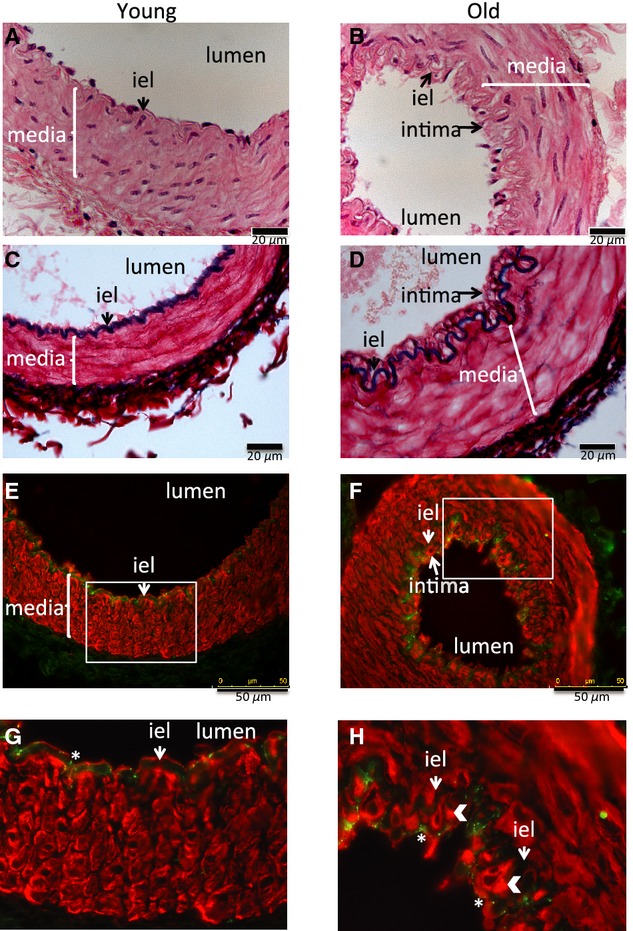
Age‐related intima formation. Representative sections of mesenteric artery stained with hematoxylin and eosin (A and B), Miller reagent for elastin (C and D), von Willebrand Factor (green) for endothelial cells, and *α*‐smooth muscle actin (red) for smooth muscle cells (E–H) from (A, C, E, and G) young animal and (B, D, F, and H) old animal. (G and H) Enlargement of the areas within the white box in (E and F). White arrow heads in (H) show intimal *α*‐smooth cell actin‐positive cells, * in G and H show von Willebrand staining in endothelial cells, “iel” internal elastic lamina. A–D scale bar 20 *μ*m, E and F scale bar 50 *μ*m.

To investigate whether extracellular remodeling of the artery wall occurred with aging the elastin and collagen contents were determined by histological measurements of sections of small arteries, fixed under passive conditions. Representative sections from young and old animals are shown in Figure 2A and B. Total collagen was greater in MSA from old compared to young animals (Fig. 2D). In contrast no age‐related differences in elastin content were detected (Fig. 2Ai, Bi, and C) resulting in reduced elastin/collagen ratio (E/C) in aged arteries (Fig. 2E). Additionally, histological analysis showed that there was a difference in the distribution and amount of collagen in MSA from young and old animals. Collagen was detected in the intima of MSA from old animals but not young, although it was present in the adventitia in both age groups (Fig. 2Aii, iii and Bii, iii). Histological staining of artery sections showed that in arteries from five of the seven old animals there was a thickened intimal cell layer on the luminal side of the internal elastic lamina. There was no neointima present in any of the arteries from the seven control animals studied. Representative sections from each group are shown in Figure 3A–D. To identify the cell types present within the neointima, sections from three young and three old animals were double stained with vWF and *α*‐sma, endothelial and smooth muscle cell markers, respectively (Fig. 3E–H). Positive staining for *α*‐sma was found within individual cells in the neointima of the arteries from old animals (Fig. 3F and H), demonstrating the presence of vascular smooth muscle cells. von Willebrand factor staining was limited to cells lining the lumen suggesting an intact endothelial cell layer in arteries from both young and old animals (Fig. 3E–H).

### Small artery mechanical properties

**Figure 4.**
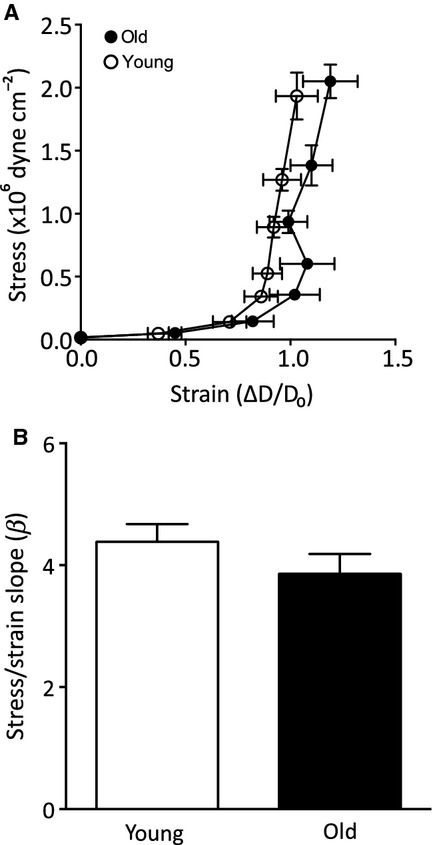
Small artery mechanical properties. (A) Stress–strain relationship and (B) *β* value, measure of arterial distensibility, in mesenteric small arteries from young (open symbols) and old (closed symbols) sheep in calcium‐free physiological salt solution. Data were calculated as described in Methods section. Data are expressed as mean ± SEM from individual arteries from eight young and five old animals.

To determine whether the age‐related artery remodeling and decrease in the elastin/collagen ratio resulted in altered stiffness, we studied the mechanical properties of the MSA. The stress–strain relationship of MSA from old animals was shifted to the right of that seen for the young animals, indicating an increased distensibility (Fig. 4A). However, the *β* values were not significantly different (Fig. 4B). The lack of an increase in stiffness in the old MSA compared to young indicates that the increased collagen observed in the old arteries does not affect their gross mechanical properties.

### Age‐related activation of sphingomyelinases

**Figure 5.**
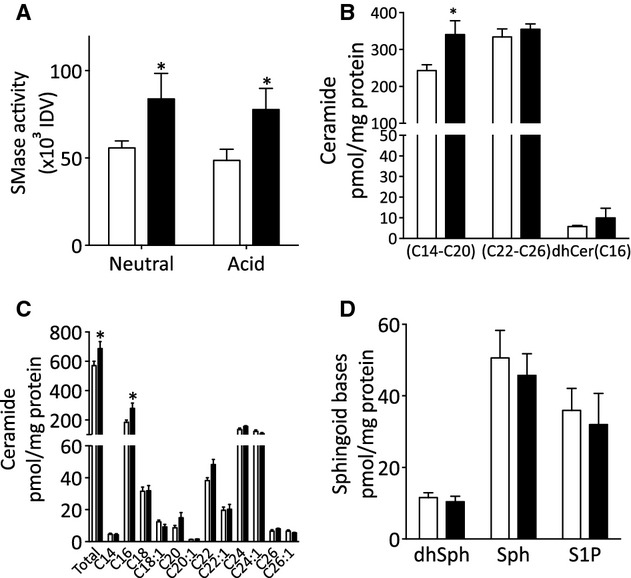
Sphingomyelinase activity and sphingolipid profile of mesenteric small arteries. (A) Neutral and acid sphingomyelinase activity measured in lysates from mesenteric arteries as described in Methods from five young (open bars) and five old (closed bars) sheep. (B, C, and D) Mass spectroscopy analysis of sphingolipids from mesenteric small arteries; (B) long‐chain (C14–C20), very long‐chain (C22–C26) ceramide, and dihydroC16‐ceramide (dhCer[C16]) levels; (C) total ceramide levels and ceramide species profile; (D) sphingoid base levels, dihydrosphingosine (dhSph), sphingosine (Sph), and sphingosine‐1‐phosphate (S1P). Data are expressed as mean ± SEM from individual arteries from four young (open bars) and three old (closed bars) animals, *P <**0.05 by (A) Student's *t*‐test, (B and C) ANOVA.

Changes in sphingolipid metabolism are a hallmark of aging implicated in age‐related tissue remodeling (Dhami et al. 2010; Moles et al. 2010). Sphingomyelinase activity assays demonstrated that there was approximately 50% greater neutral and acid sphingomyelinase activity in MSA from old compared to young animals (Fig. 5A). To determine whether the increased sphingomyelinase activity resulted in altered ceramide levels, sphingolipids were quantified from MSA homogenates of young and old animals. There was an approximately 40% increase in long‐chain ceramide (C14–C20), but no change in very long‐chain ceramide (C22–C26; Fig. 5B). The increase in long‐chain ceramide (C14–C20) was due to a marked increase in C16‐ceramide (Fig. 5C). Dihydroceramide (C16), the precursor of ceramide in the de novo synthesis pathway was not altered between young and old MSA (Fig. 5B) indicating that the origin of the long‐chain ceramide was from sphingomyelin hydrolysis by sphingomyelinases. Additionally, the sphingoid bases dihydrosphingosine, sphingosine, and sphingosine‐1‐phosphate were not altered with aging (Fig. 5D) suggesting there was no increased metabolism of ceramide through this pathway in arteries from old animals.

### Small artery reactivity

**Figure 6.**
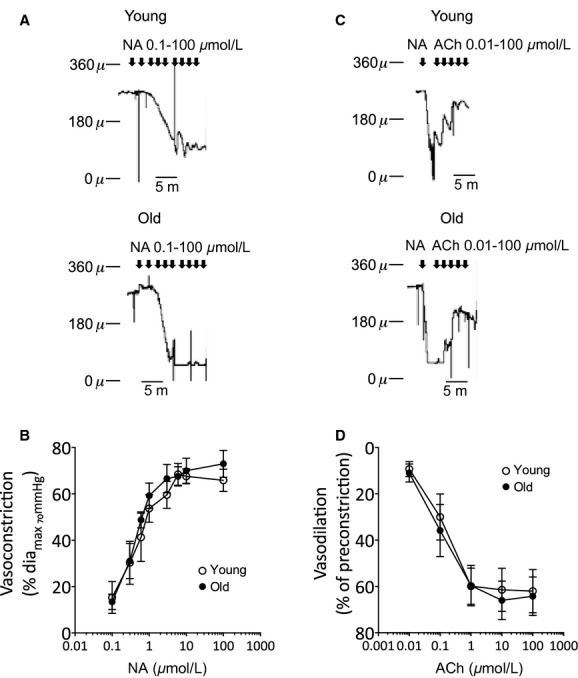
Small artery contractility. Mesenteric small arteries were mounted in a pressure myograph and cumulative responses to noradrenaline (NA) or acetylcholine (ACh) obtained as detailed in Methods. (A) Representative tracings showing contractile response to noradrenaline in an artery from a young animal (upper panel) and an old animal (lower panel), (B) average data of the cumulative concentration response to NA, data are normalized to the passive diameter at 70 mmHg as detailed in Methods, (C) representative tracings showing relaxation response to ACh in arteries preconstricted with NA (15 *μ*mol/L), *upper panel*: an artery from a young animal and *lower panel*: an old animal; (D) average data of the cumulative concentration response to ACh, data are normalized to the preconstricted diameter as detailed in Methods. Data are expressed as mean ± SEM from individual arteries from five young (open circle) and five old (closed circle) animals.

Remodeling of small arteries may lead to changes in their reactivity to vasoconstrictor stimuli and/or endothelial function (Martinez‐Lemus et al. 2009). To investigate this we studied the response of MSA from young and aged animals to noradrenaline and acetylcholine. There was no difference in either the sensitivity or maximal contractile response to noradrenaline in MSA from old compared to young animals (Fig. 6A). Nor was there any difference in the relaxation of preconstricted arteries to acetylcholine (Fig. 6B) indicating that there was no age‐related change in endothelium‐dependent vasodilation.

## Discussion

In this study, we found increased sphingomyelinase activity and accumulation of long‐chain ceramides in small arteries from old sheep compared to young. Additionally, we observed an increase in total collagen content in arteries from old animals. Ceramide promotes collagen deposition in dermal fibroblasts (Sato et al. 2003) implicating this lipid in the extracellular matrix remodeling of small arteries in age.

Investigation of the morphology and mechanical properties of small arteries showed that under passive conditions the mesenteric small arteries from old sheep had increased lumen diameter and media thickness without a change in media to lumen ratio, indicative of outward hypertrophic remodeling (Mulvany 1999). In addition, there was deposition of collagen in a thickened intima layer that contained both endothelial and smooth muscle cells in arteries from old animals. However, despite increased collagen content and decreased elastin to collagen ratio there was no change in stiffness in the aged sheep arteries. This is in contrast to studies in rats where age‐related increases in mesenteric and cerebral small artery stiffness are reported (Hajdu et al. 1990; Adrian et al. 2004; Laurant et al. 2004; Briones et al. 2007). However, it is in agreement with studies in humans where elastic modulus and compliance of small arteries also change very little with age (McEniery et al. 2007). Although age‐related increased stiffness of resistance arteries from rats has been reported (Hajdu et al. 1990; Adrian et al. 2004; Laurant et al. 2004; Briones et al. 2007), there is little evidence to support that collagen content correlates with small artery stiffness or distensibility (Izzard et al. 2003; González et al. 2005; Mandalà et al. 2012). Indeed, an age‐related change in elastin fiber organization appears to be a more important determinant of resistance artery stiffness (González et al. 2005). In our study we did not examine elastic fiber integrity, but in the aorta from aged sheep there was no change in stiffness associated with the elastic lamellae (Graham et al. 2011) suggesting elastic fiber structure does not alter with aging in this animal model. This is further supported by our observation that the *β* value, a measure of distensibility independent of artery wall geometry (Izzard et al. 2006), that reflects the combined elastic moduli of the elastic components of the artery wall did not change in small arteries with age. No change in arterial distensibility in the presence of remodeling has been reported also in rat small arteries with age (Adrian et al. 2004; Laurant et al. 2004; Mandalà et al. 2012). This suggests that preservation of distensibility is physiologically important and may be actively regulated in small arteries. Although large artery stiffness positively correlates with age, urban lifestyle, and pathological conditions such as hypertension, inflammation, and diabetes are known to accelerate the stiffening process (Avolio et al. 1985; Lemogoum et al. 2012; Wilkinson and McEniery 2012). The old animals used in our study did not have increased systolic blood pressure, pulse pressure, or heart rate compared to young animals nor were there any alterations in the reactivity of the arteries. This shows that the remodeling we found in small arteries is related to age and that increased arterial stiffness is not an inevitable consequence of aging.

Alterations in vascular contraction and relaxation have been reported with aging. The majority of studies have been conducted on large conduit arteries and mainly demonstrated loss of endothelial‐dependent relaxation (reviewed in Wang et al. 2010). However, in resistance arteries there is little evidence of altered vascular reactivity in arteries from rodent models of aging (Cook et al. 1992; Moreau et al. 1998; Gros et al. 2002; Muller‐Delp et al. 2002; Mandalà et al. 2012) or human vessels (Nyborg and Nielsen 1990). Our study in a large animal model also showed that despite small artery remodeling there was no change in the contractile response to NA or to ACh‐induced relaxation. It is unlikely that these negative results were due to the relatively small group sizes because we detected no trends toward altered responsiveness even when the data were normalized to account for differences in passive diameters between the two groups. These data suggest that in healthy aging any changes that occur in hemodynamics in the mesenteric resistance circulation will reflect remodeling changes rather than altered reactivity. However, caution must be exercised when inferring hemodynamic changes in vivo from structural changes observed in vitro. Additionally, due to availability of animals our study used female animals only. Therefore, whether the changes we have observed occur in males also or are specific to females is not known. Furthermore, recent evidence suggests there is an age‐related decline in the ability of small arteries to sense changes in intraluminal pressure resulting in impaired myogenic responsiveness and poor autoregulation of blood flow (Nyborg and Nielsen 1990; Gros et al. 2002; Muller‐Delp et al. 2002). This is not accompanied by a decrease in contraction to vasoconstrictors (Cook et al. 1992; Moreau et al. 1998; Gros et al. 2002; Muller‐Delp et al. 2002), indicating that the defect is due to a change in the ability of vascular smooth muscle cells to sense and respond to changes in blood pressure, that is impaired mechanotransduction. However, although we did not investigate myogenic responses of the arteries in our study, we found no evidence of differences in basal myogenic tone between arteries from young and old animals (active diameter at 70 mmHg as percentage of passive diameter at 70 mmHg: young, 4.67 ± 2.78; old, 7.27 ± 1.20; *P* = 0.21), indicating minimal basal tone in our experiments. This is perhaps not unexpected given that mesenteric arteries are less myogenic than for instance cerebral arteries. Recently S1P has been identified as a mediator of myogenic tone in hamster arteries and in mouse mesenterics in heart failure (Bolz et al. 2003; Hoefer et al. 2010). However, our lipid measurements showed that there was no change in sphingoid bases (sphingosine and S1P) in the arteries from old animals compared to young, indicating that there was no increase in the flux of ceramide to S1P in the aging tissues. This further supports that in the conditions used in our study there was no marked difference in myogenicity between the groups.

Alterations in cellular sphingolipids appear to be a hallmark of aging. An age‐related increase in basal ceramide levels has been found in diverse tissues including rat liver and brain (Lightle et al. 2000; Cutler et al. 2004), mouse kidney, and human fibroblasts (Hernández‐Corbacho et al. 2011). Our data show that in sheep MSA there was an age‐related increase in long‐chain ceramide (C14–C20). Although ceramide is the precursor of all sphingolipids and as such acts as a metabolic hub (Hannun and Obeid 2011) being readily converted to sphingosine and S1P, our observation that only ceramide accumulated in small arteries from old sheep suggests that ceramide is the key player in age‐related changes in this tissue. Evidence is now accumulating that individual species of ceramide mediate specific cellular responses (Hannun and Obeid 2011). We detected mainly an increase in C16‐ceramide, this ceramide species has been implicated in apoptosis and cell death (Hannun and Obeid 2011) although whether C16‐ceramide plays a specific role in aging is not known. The increase in ceramide was paralleled by an increase in both acid‐ and neutral‐sphingomyelinase activity. There is evidence that both acid and neutral sphingomyelinase activity is increased with age (Lecka‐Czernik et al. 1996; Nikolova‐Karakashian et al. 2008). Relevant to the vasculature, increased acid sphingomyelinase expression and ceramide accumulation is associated with premature senescence in endothelial cells in vitro (Patschan et al. 2008). However, we found no alteration in endothelial‐dependent relaxation of MSA from old animals compared to young suggesting that in small arteries endothelial cell function was not impaired with aging.

Ceramide is implicated in many responses that could contribute to age‐related remodeling of the vasculature. For instance, ceramide is a major signaling molecule in cellular responses to stress and regulates apoptosis, senescence, and fibrosis (Ogretmen and Hannun 2004). Ceramide promotes collagen deposition in keratinocytes in vitro (Sato et al. 2003) and increased A‐SMase activity and elevated ceramide levels are involved in lung and liver fibrosis in vivo (Dhami et al. 2010; Moles et al. 2010). Consequently, alterations in any of these processes could lead to remodeling of the artery wall. Additionally, ceramide regulates actin cytoskeleton dynamics in breast cancer cells through modulation of ezrin, radixin, moesin function (Zeidan et al. 2008). Altered actin cytoskeleton dynamics have been reported in vascular smooth muscle cells from large arteries with age (Li et al. 1997; Qiu et al. 2010). The actin cytoskeleton is a vital component of vascular smooth muscle cell mechanosensing (Hill and Meininger 2012) and the myogenic response (Walsh and Cole 2013), suggesting ceramide could also play a role in impaired mechanotransduction in small arteries with aging. However, to begin to understand the mechanisms of sphingolipid actions in the age‐related changes in small arteries more fully, it is first necessary to identify the sphingolipid metabolizing enzymes and the site of ceramide production in vascular tissues.

In summary, this study demonstrates remodeling of small arteries during aging that is accompanied by increased sphingomyelinase activity and accumulation of long‐chain ceramides. This suggests sphingolipids maybe important mediators of vascular aging. Given that aging is a major risk factor for cardiovascular disease, our study opens a new area for further research into the mechanisms that underlie vascular remodeling in aging.

## Acknowledgments

We are grateful to Professor Andrew W Trafford, Institute of Cardiovascular Sciences, University of Manchester, Manchester, UK for providing tissue from young and old sheep.

## Conflict of Interest

None declared.

## References

[b1] Adrian M. Laurant P. Berthelot A. 2004 Effect of magnesium on mechanical properties of pressurized mesenteric small arteries from old and adult rats. Clin. Exp. Pharmacol. Physiol.; 31:306-313 15191403 10.1111/j.1440-1681.2004.03992.x

[b2] Akopov E. S. Zhang L. Pearce W. J. 1998 Regulation of Ca sensitization by PKC and rho proteins in ovine cerebral arteries: effects of artery size and age. Am. J. Physiol.; 275:H930-H939 9724297 10.1152/ajpheart.1998.275.3.H930

[b3] Alewijnse A. E. Peters S. L. M. 2008 Sphingolipid signalling in the cardiovascular system: good, bad or both? Eur. J. Pharmacol.; 585:292-302 18420192 10.1016/j.ejphar.2008.02.089

[b4] Avolio A. P. Deng F. Q. Li W. Q. Luo Y. F. Huang Z. D. Xing L. F. 1985 Effects of aging on arterial distensibility in populations with high and low prevalence of hypertension: comparison between urban and rural communities in China. Circulation; 71:202-210 3965165 10.1161/01.cir.71.2.202

[b5] Bielawski J. Pierce J. S. Snider J. Rembiesa B. Szulc Z. M. Bielawska A. 2010 Sphingolipid analysis by high performance liquid chromatography‐tandem mass spectrometry (HPLC‐MS/MS). Adv. Exp. Med. Biol.; 688:46-59 20919645 10.1007/978-1-4419-6741-1_3

[b6] Bolz S.‐S. Vogel L. Sollinger D. Derwand R. Boer C. Pitson S. M. 2003 Sphingosine kinase modulates microvascular tone and myogenic responses through activation of RhoA/Rho kinase. Circulation; 108:342-347 12847068 10.1161/01.CIR.0000080324.12530.0D

[b7] Briones A. M. Salaices M. Vila E. 2007 Mechanisms underlying hypertrophic remodeling and increased stiffness of mesenteric resistance arteries from aged rats. J. Gerontol. A Biol. Sci. Med. Sci.; 62:696-706 17634315 10.1093/gerona/62.7.696

[b8] Clarke C. J. Forman S. Pritchett J. Ohanian V. Ohanian J. 2008 Phospholipase C‐delta1 modulates sustained contraction of rat mesenteric small arteries in response to noradrenaline, but not endothelin‐1. Am. J. Physiol. Heart Circ. Physiol.; 295:H826-H834 18567701 10.1152/ajpheart.01396.2007 PMC2519204

[b9] Cook J. J. Wailgum T. D. Vasthare U. S. Mayrovitz H. N. Tuma R. F. 1992 Age‐related alterations in the arterial microvasculature of skeletal muscle. J. Gerontol.; 47:B83-B88 1573183 10.1093/geronj/47.3.b83

[b10] Cutler R. G. Kelly J. Storie K. Pedersen W. A. Tammara A. Hatanpaa K. 2004 Involvement of oxidative stress‐induced abnormalities in ceramide and cholesterol metabolism in brain aging and Alzheimer's disease. Proc. Natl. Acad. Sci. USA; 101:2070-2075 14970312 10.1073/pnas.0305799101 PMC357053

[b11] Dhami R. He X. Schuchman E. H. 2010 Acid sphingomyelinase deficiency attenuates bleomycin‐induced lung inflammation and fibrosis in mice. Cell. Physiol. Biochem.; 26:749-760 21063112 10.1159/000322342 PMC3048941

[b12] Dibb K. M. Rueckschloss U. Eisner D. A. Isenberg G. Trafford A. W. 2004 Mechanisms underlying enhanced cardiac excitation contraction coupling observed in the senescent sheep myocardium. J. Mol. Cell. Cardiol.; 37:1171-1181 15572047 10.1016/j.yjmcc.2004.09.005

[b13] D'Mello N. P. Childress A. M. Franklin D. S. Kale S. P. Pinswasdi C. Jazwinski S. M. 1994 Cloning and characterization of LAG1, a longevity‐assurance gene in yeast. J. Biol. Chem.; 269:15451-15459 8195187

[b14] Flamm S. D. Taki J. Moore R. Lewis S. F. Keech F. Maltais F. 1990 Redistribution of regional and organ blood volume and effect on cardiac function in relation to upright exercise intensity in healthy human subjects. Circulation; 81:1550-1559 2331767 10.1161/01.cir.81.5.1550

[b15] Franceschi C. Bonafè M. Valensin S. Olivieri F. De Luca M. Ottaviani E. 2000 Inflamm‐aging. An evolutionary perspective on immunosenescence. Ann. N. Y. Acad. Sci.; 908:244-254 10911963 10.1111/j.1749-6632.2000.tb06651.x

[b16] González J. M. Briones A. M. Starcher B. Conde M. V. Somoza B. Daly C. 2005 Influence of elastin on rat small artery mechanical properties. Exp. Physiol.; 90:463-468 15890799 10.1113/expphysiol.2005.030056

[b17] Graham H. K. Trafford A. W. 2007 Spatial disruption and enhanced degradation of collagen with the transition from compensated ventricular hypertrophy to symptomatic congestive heart failure. Am. J. Physiol. Heart Circ. Physiol.; 292:H1364-H1372 17071734 10.1152/ajpheart.00355.2006

[b18] Graham H. K. Akhtar R. Kridiotis C. Derby B. Kundu T. Trafford A. W. 2011 Localised micro‐mechanical stiffening in the ageing aorta. Mech. Ageing Dev.; 132:459-467 21777602 10.1016/j.mad.2011.07.003 PMC3192262

[b19] Greenwald S. E. 2007 Ageing of the conduit arteries. J. Pathol.; 211:157-172 17200940 10.1002/path.2101

[b20] Gros R. Van Wert R. You X. Thorin E. Husain M. 2002 Effects of age, gender, and blood pressure on myogenic responses of mesenteric arteries from C57BL/6 mice. Am. J. Physiol. Heart Circ. Physiol.; 282:H380-H388 11748085 10.1152/ajpheart.2002.282.1.H380

[b21] Guillas I. Kirchman P. A. Chuard R. Pfefferli M. Jiang J. C. Jazwinski S. M. 2001 C26‐CoA‐dependent ceramide synthesis of Saccharomyces cerevisiae is operated by Lag1p and Lac1p. EMBO J.; 20:2655-2665 11387200 10.1093/emboj/20.11.2655 PMC125493

[b22] Hajdu M. A. Heistad D. D. Siems J. E. Baumbach G. L. 1990 Effects of aging on mechanics and composition of cerebral arterioles in rats. Circ. Res.; 66:1747-1754 2344672 10.1161/01.res.66.6.1747

[b23] Hannun Y. A. Obeid L. M. 2008 Principles of bioactive lipid signalling: lessons from sphingolipids. Nat. Rev. Mol. Cell Biol.; 9:139-150 18216770 10.1038/nrm2329

[b24] Hannun Y. A. Obeid L. M. 2011 Many ceramides. J. Biol. Chem.; 286:27855-27862 21693702 10.1074/jbc.R111.254359 PMC3151029

[b25] Hernández‐Corbacho M. J. Jenkins R. W. Clarke C. J. Hannun Y. A. Obeid L. M. Snider A. J. 2011 Accumulation of long‐chain glycosphingolipids during aging is prevented by caloric restriction. PLoS One; 6:e20411 21687659 10.1371/journal.pone.0020411 PMC3110726

[b26] Herrera E. A. Riquelme R. A. Ebensperger G. Reyes R. V. Ulloa C. E. Cabello G. 2010 Long‐term exposure to high‐altitude chronic hypoxia during gestation induces neonatal pulmonary hypertension at sea level. Am. J. Physiol. Regul. Integr. Comp. Physiol.; 299:R1676-R1684 20881096 10.1152/ajpregu.00123.2010 PMC3007194

[b27] Hill M. A. Meininger G. A. 2012 Arteriolar vascular smooth muscle cells: mechanotransducers in a complex environment. Int. J. Biochem. Cell Biol.; 44:1505-1510 22677491 10.1016/j.biocel.2012.05.021 PMC4221252

[b28] Hoefer J. Azam M. A. Kroetsch J. T. E. Leong‐Poi H. Momen M. A. Voigtlaender‐Bolz J. 2010 Sphingosine‐1‐phosphate‐dependent activation of p38 MAPK maintains elevated peripheral resistance in heart failure through increased myogenic vasoconstriction. Circ. Res.; 107:923-933 20671234 10.1161/CIRCRESAHA.110.226464

[b29] Horn M. A. Graham H. K. Richards M. A. Clarke J. D. Greensmith D. J. Briston S. J. 2012 Age‐related divergent remodeling of the cardiac extracellular matrix in heart failure: Collagen accumulation in the young and loss in the aged. J. Mol. Cell. Cardiol.; 53:82-90 22516365 10.1016/j.yjmcc.2012.03.011

[b30] Izzard A. S. Graham D. Burnham M. P. Heerkens E. H. Dominiczak A. F. Heagerty A. M. 2003 Myogenic and structural properties of cerebral arteries from the stroke‐prone spontaneously hypertensive rat. Am. J. Physiol. Heart Circ. Physiol.; 285:H1489-H1494 12816753 10.1152/ajpheart.00352.2003

[b31] Izzard A. S. Horton S. Heerkens E. H. Shaw L. Heagerty A. M. 2006 Middle cerebral artery structure and distensibility during developing and established phases of hypertension in the spontaneously hypertensive rat. J. Hypertens.; 24:875-880 16612249 10.1097/01.hjh.0000222757.54111.06

[b32] Lakatta E. G. Levy D. 2003 Arterial and cardiac aging: major shareholders in cardiovascular disease enterprises: part I: aging arteries: a “set up” for vascular disease. Circulation; 107:139-146 12515756 10.1161/01.cir.0000048892.83521.58

[b33] Laurant P. Adrian M. Berthelot A. 2004 Effect of age on mechanical properties of rat mesenteric small arteries. Can. J. Physiol. Pharmacol.; 82:269-275 15181465 10.1139/y04-026

[b34] Lecka‐Czernik B. Moerman E. J. Jones R. A. Goldstein S. 1996 Identification of gene sequences overexpressed in senescent and Werner syndrome human fibroblasts. Exp. Gerontol.; 31:159-174 8706786 10.1016/0531-5565(95)02014-4

[b35] Lemogoum D. Ngatchou W. Janssen C. Leeman M. Van Bortel L. Boutouyrie P. 2012 Effects of hunter‐gatherer subsistence mode on arterial distensibility in Cameroonian pygmies. Hypertension; 60:123-128 22615114 10.1161/HYPERTENSIONAHA.111.187757

[b36] Li Z. Cheng H. Lederer W. J. Froehlich J. Lakatta E. G. 1997 Enhanced proliferation and migration and altered cytoskeletal proteins in early passage smooth muscle cells from young and old rat aortic explants. Exp. Mol. Pathol.; 64:1-11 9203504 10.1006/exmp.1997.2204

[b37] Lightle S. A. Oakley J. I. Nikolova‐Karakashian M. N. 2000 Activation of sphingolipid turnover and chronic generation of ceramide and sphingosine in liver during aging. Mech. Ageing Dev.; 120:111-125 11087909 10.1016/s0047-6374(00)00191-3

[b38] Loidl A. Claus R. Deigner H. P. Hermetter A. 2002 High‐precision fluorescence assay for sphingomyelinase activity of isolated enzymes and cell lysates. J. Lipid Res.; 43:815-823 11971953

[b39] Mandalà M. Pedatella A. L. Morales Palomares S. Cipolla M. J. Osol G. 2012 Maturation is associated with changes in rat cerebral artery structure, biomechanical properties and tone. Acta Physiol. (Oxf); 205:363-371 22212496 10.1111/j.1748-1716.2011.02406.x

[b40] Martinez‐Lemus L. A. Hill M. A. Meininger G. A. 2009 The plastic nature of the vascular wall: a continuum of remodeling events contributing to control of arteriolar diameter and structure. Physiology (Bethesda); 24:45-57 19196651 10.1152/physiol.00029.2008

[b41] McEniery C. M. Wilkinson I. B. Avolio A. P. 2007 Age, hypertension and arterial function. Clin. Exp. Pharmacol. Physiol.; 34:665-671 17581227 10.1111/j.1440-1681.2007.04657.x

[b42] McEniery C. M. Yasmin Maki‐Petaja K. M. McDonnell B. J. Munnery M. Hickson S. S. Franklin S. S. 2010 The impact of cardiovascular risk factors on aortic stiffness and wave reflections depends on age: the Anglo‐Cardiff Collaborative Trial (ACCT III). Hypertension; 56:591-597 20696989 10.1161/HYPERTENSIONAHA.110.156950

[b43] Moles A. Tarrats N. Morales A. Domínguez M. Bataller R. Caballería J. 2010 Acidic sphingomyelinase controls hepatic stellate cell activation and in vivo liver fibrogenesis. Am. J. Pathol.; 177:1214-1224 20651240 10.2353/ajpath.2010.091257 PMC2928955

[b44] Moreau P. d'Uscio L. V. Lüscher T. F. 1998 Structure and reactivity of small arteries in aging. Cardiovasc. Res.; 37:247-253 9539880 10.1016/s0008-6363(97)00225-3

[b45] Muller‐Delp J. Spier S. A. Ramsey M. W. Lesniewski L. A. Papadopoulos A. Humphrey J. D. 2002 Effects of aging on vasoconstrictor and mechanical properties of rat skeletal muscle arterioles. Am. J. Physiol. Heart Circ. Physiol.; 282:H1843-H1854 11959651 10.1152/ajpheart.00666.2001

[b46] Mulvany M. 1999 Vascular remodelling of resistance vessels: can we define this? Cardiovasc. Res.; 41:9-13 10325946 10.1016/s0008-6363(98)00289-2

[b47] Nikolova‐Karakashian M. Karakashian A. Rutkute K. 2008 Role of neutral sphingomyelinases in aging and inflammation. Subcell. Biochem.; 49:469-486 18751923 10.1007/978-1-4020-8831-5_18

[b48] Nyborg N. C. Nielsen P. J. 1990 The level of spontaneous myogenic tone in isolated human posterior ciliary arteries decreases with age. Exp. Eye Res.; 51:711-715 2265682 10.1016/0014-4835(90)90056-z

[b49] Obeid L. M. Hannun Y. A. 2003 Ceramide, stress, and a “LAG” in aging. Sci. Aging Knowledge Environ.; 2003:PE27 14523222 10.1126/sageke.2003.39.pe27

[b50] Ogretmen B. Hannun Y. A. 2004 Biologically active sphingolipids in cancer pathogenesis and treatment. Nat. Rev. Cancer; 4:604-616 15286740 10.1038/nrc1411

[b51] Ohanian J. Forman S. P. Katzenberg G. Ohanian V. 2012 Endothelin‐1 stimulates small artery VCAM‐1 expression through p38MAPK‐dependent neutral sphingomyelinase. J. Vasc. Res.; 49:353-362 22627111 10.1159/000336649

[b52] Patschan S. Chen J. Polotskaia A. Mendelev N. Cheng J. Patschan D. 2008 Lipid mediators of autophagy in stress‐induced premature senescence of endothelial cells. Am. J. Physiol. Heart Circ. Physiol.; 294:H1119-H1129 18203850 10.1152/ajpheart.00713.2007

[b53] Qiu H. Zhu Y. Sun Z. Trzeciakowski J. P. Gansner M. Depre C. 2010 Short communication: vascular smooth muscle cell stiffness as a mechanism for increased aortic stiffness with aging. Circ. Res.; 107:615-619 20634486 10.1161/CIRCRESAHA.110.221846 PMC2936100

[b54] Rao R. P. Yuan C. Allegood J. C. Rawat S. S. Edwards M. B. Wang X. 2007 Ceramide transfer protein function is essential for normal oxidative stress response and lifespan. Proc. Natl. Acad. Sci. USA; 104:11364-11369 17592126 10.1073/pnas.0705049104 PMC1899189

[b55] Rowell L. B. 1974 Human cardiovascular adjustments to exercise and thermal stress. Physiol. Rev.; 54:75-159 4587247 10.1152/physrev.1974.54.1.75

[b56] Sato M. Markiewicz M. Yamanaka M. Bielawska A. Mao C. Obeid L. M. 2003 Modulation of transforming growth factor‐beta (TGF‐beta) signaling by endogenous sphingolipid mediators. J. Biol. Chem.; 278:9276-9282 12515830 10.1074/jbc.M211529200

[b57] Schorling S. Vallée B. Barz W. P. Riezman H. Oesterhelt D. 2001 Lag1p and Lac1p are essential for the Acyl‐CoA‐dependent ceramide synthase reaction in Saccharomyces cerevisae. Mol. Biol. Cell; 12:3417-3427 11694577 10.1091/mbc.12.11.3417 PMC60264

[b58] Smith A. R. Visioli F. Frei B. Hagen T. M. 2006 Age‐related changes in endothelial nitric oxide synthase phosphorylation and nitric oxide dependent vasodilation: evidence for a novel mechanism involving sphingomyelinase and ceramide‐activated phosphatase 2A. Aging Cell; 5:391-400 16930126 10.1111/j.1474-9726.2006.00232.x

[b59] Ungvari Z. Kaley G. De Cabo R. Sonntag W. E. Csiszar A. 2010 Mechanisms of vascular aging: new perspectives. J. Gerontol. A Biol. Sci. Med. Sci.; 65:1028-1041 20576649 10.1093/gerona/glq113 PMC2950814

[b60] Venable M. E. Yin X. 2009 Ceramide induces endothelial cell senescence. Cell Biochem. Funct.; 27:547-551 19842094 10.1002/cbf.1605

[b61] Walsh M. P. Cole W. C. 2013 The role of actin filament dynamics in the myogenic response of cerebral resistance arteries. J. Cereb. Blood Flow Metab.; 33:1-12 23072746 10.1038/jcbfm.2012.144 PMC3597360

[b62] Wang M. Monticone R. E. Lakatta E. G. 2010 Arterial aging: a journey into subclinical arterial disease. Curr. Opin. Nephrol. Hypertens.; 19:201-207 20040868 10.1097/MNH.0b013e3283361c0b PMC2943205

[b63] Wilkinson I. B. McEniery C. M. 2012 Arteriosclerosis: inevitable or self‐inflicted? Hypertension; 60:3-5 22615110 10.1161/HYPERTENSIONAHA.112.193029

[b64] Williams J. M. Pearce W. J. 2006 Age‐dependent modulation of endothelium‐dependent vasodilatation by chronic hypoxia in ovine cranial arteries. Int. J. Appl. Physiol.; 100:225-232 10.1152/japplphysiol.00221.2005 16179402

[b65] Xiao D. Huang X. Yang S. Longo L. D. Zhang L. 2010 Pregnancy downregulates actin polymerization and pressure‐dependent myogenic tone in ovine uterine arteries. Hypertension; 56:1009-1015 20855655 10.1161/HYPERTENSIONAHA.110.159137 PMC3001123

[b66] Yamanaka M. Shegogue D. Pei H. Bu S. Bielawska A. Bielawski J. 2004 Sphingosine kinase 1 (SPHK1) is induced by transforming growth factor‐beta and mediates TIMP‐1 up‐regulation. J. Biol. Chem.; 279:53994-54001 15485866 10.1074/jbc.M410144200

[b67] Zeidan Y. H. Jenkins R. W. Hannun Y. A. 2008 Remodeling of cellular cytoskeleton by the acid sphingomyelinase/ceramide pathway. J. Cell Biol.; 181:335-350 18426979 10.1083/jcb.200705060 PMC2315679

